# ROS Homeostasis Regulates Somatic Embryogenesis *via* the Regulation of Auxin Signaling in Cotton* [Fn FN2]

**DOI:** 10.1074/mcp.M115.049338

**Published:** 2016-04-12

**Authors:** Ting Zhou, Xiyan Yang, Kai Guo, Jinwu Deng, Jiao Xu, Wenhui Gao, Keith Lindsey, Xianlong Zhang

**Affiliations:** From the ‡National Key Laboratory of Crop Genetic Improvement, Huazhong Agricultural University, Wuhan, Hubei 430070, P. R. China;; §Integrative Cell Biology Laboratory, School of Biological and Biomedical Sciences, University of Durham, South Road, Durham DH1 3LE, United Kingdom

## Abstract

Somatic embryogenesis (S.E.) is a versatile model for understanding the mechanisms of plant embryogenesis and a useful tool for plant propagation. To decipher the intricate molecular program and potentially to control the parameters affecting the frequency of S.E., a proteomics approach based on two-dimensional gel electrophoresis (2-DE) combined with MALDI-TOF/TOF was used. A total of 149 unique differentially expressed proteins (DEPs) were identified at different stages of cotton S.E. compared with the initial control (0 h explants). The expression profile and functional annotation of these DEPs revealed that S.E. activated stress-related proteins, including several reactive oxygen species (ROS)-scavenging enzymes. Proteins implicated in metabolic, developmental, and reproductive processes were also identified. Further experiments were performed to confirm the role of ROS-scavenging enzymes, suggesting the involvement of ROS homeostasis during S.E. in cotton. Suppressing the expression of specifically identified GhAPX proteins resulted in the inhibition of dedifferentiation. Accelerated redifferentiation was observed in the suppression lines of GhAPXs or GhGSTL3 in parallel with the alteration of endogenous ascorbate metabolism and accumulation of endogenous H_2_O_2_ content. Moreover, disrupting endogenous redox homeostasis through the application of high concentrations of DPI, H_2_O_2_, BSO, or GSH inhibited the dedifferentiation of cotton explants. Mild oxidation induced through BSO treatment facilitated the transition from embryogenic calluses (ECs) to somatic embryos. Meanwhile, auxin homeostasis was altered through the perturbation of ROS homeostasis by chemical treatments or suppression of ROS-scavenging proteins, along with the activating/suppressing the transcription of genes related to auxin transportation and signaling. These results show that stress responses are activated during S.E. and may regulate the ROS homeostasis by interacting with auxin signaling.

During somatic embryogenesis (S.E.), differentiated somatic cells reentering the cell cycle undergo dedifferentiation and redifferentiation, followed by the formation of embryogenic cells and somatic embryos, and eventually develop into new plants (1). Such developmental restructuring involves the orchestration of various signal networks and reprogramming gene expression patterns to alter the original development program (2, 3). The observed totipotency of somatic cells, allowing them to initiate embryogenic development under appropriate conditions, has been exploited to explore regulatory mechanisms and morphogenetic events occurring during the initiation and development of plant embryogenesis and represents a powerful tool for the propagation of plants combined with genetic engineering (1, 3, 4).

Recent analysis of the genes implicated in S.E. or exhibiting differential expression during S.E. were performed to uncover the molecular events of S.E (5–7). Although the identification of these genes increases the current understanding of embryogenic processes, the detailed mechanisms of S.E. largely remain unclear. Proteomics has emerged as a powerful tool for the systematic analysis of protein expression during particular biological processes (8). The proteomic analysis of S.E. by comparing embryogenic calluses (ECs) and nonembryogenic calluses (NECs) in different plant species has been carried out, and several differentially expressed proteins (DEPs) have been identified during S.E (9–11).

S.E. induction is a complex process affected by many factors. Progressively, research has shown that stress factors play an important role during S.E (12, 13). Reactive oxygen species (ROS) were recognized as pivotal regulators of plant growth and development (14, 15). ROS display a dual function in many developmental processes, which depend on the level and subcellular distribution of these molecules (16). High concentrations of ROS are toxic, leading to oxidative damage. However, at appropriate concentrations, ROS also act as signaling molecules regulating many developmental and physiological responses (15, 17). The modulation of ROS levels is involved in the control of cell proliferation, cell death, and senescence, particularly the destruction of subcellular organelles (16, 18). The ROS concentration and subcellular distribution in plants is carefully regulated, as imbalances cause redox state disturbances that have crucial effects on the cell fate (16). The steady state of ROS in cells is maintained through ROS-generating enzymes, such as NADPH oxidases and ROS-scavenging enzymes, including superoxide dismutases (SOD), ascorbate peroxidases (APX), catalases (CATs), glutathione peroxidase (GPX), glutathione transferase (GST), and antioxidant molecules, such as glutathione and ascorbic acid (17, 18).

ROS-mediated redox signal-regulated development is often associated with hormonal reactions and responses during plant development (19, 20). Auxin plays key roles in plant growth and developmental processes and often cross talk with ROS to modulate diverse aspects of plant growth and development (18, 21). It has been reported that indole-3-acetic acid (IAA) could be metabolized by horseradish peroxidase, and overexpression of such peroxidase in tobacco enhanced defense responses and impaired growth because of increased IAA degradation activity (22, 23). During S.E., many auxin-responsive genes are differentially expressed, and auxin-induced S.E. in cotyledons has been associated with oxidative stress and defense gene activation (6, 24). ROS generation and auxin are both required for the cell cycle progression from the G0 to the G1 phase (25). In root gravitropism, auxin induces ROS production, which requires activation of phosphatidylinositol 3-kinase, and ROS may act downstream of auxin (26, 27). Auxin also regulates the counterbalance of APX1 S-nitrosylation/denitrosylation activity to modulate APX1 activity to fine control of root development and determination of root architecture (28). Besides, ROS homeostasis can be directly modulated by auxin through inducing ROS detoxification enzymes, such as glutathione (GSH)-S-transferases, or indirectly by affecting the stability of DELLA (74) proteins (20). Reports have also shown that several redox-related genes modulate ROS crosstalk through auxin signaling (29–31). The accumulation of mitochondrial ROS in the *abo6* mutant mediated the crosstalk between ABA and auxin signaling (29). Chen *et al.* (30) revealed that *AtAPX6* mediates the crosstalk between ROS, ABA, and auxin to protect desiccating and germinating *Arabidopsis* seeds from stress. Studies on the *rbohD* and *rbohF* double mutant have shown the involvement of ROS in activating Ca^2+^ signaling and decreasing auxin sensitivity in *Arabidopsis* roots (32). The disruption of the NADP-linked thioredoxin and glutathione systems in a triple mutant elevated ROS levels and perturbed auxin transport and metabolism (33).

Cotton, as a main source of textile fiber, is one of the most important economic crops worldwide. Therefore, a reproducible and highly efficient regeneration scheme is greatly important for cotton genetic engineering (34). However, the regeneration of cotton species through S.E. was inclined to specific varieties, reflecting a genotype-dependent response (35). Thus, the underlying biochemical and molecular events during cotton S.E. remain an important research area for developmental biology. In a previous study, we identified an elite genotype, exhibiting a higher regeneration frequency than Coker lines (34). Despite the complex regulation of auxin signaling, transcription factors, miRNAs, and stress-related responses at the transcription level were investigated through next-generation sequencing of this genotype during S.E (6, 12, 36). The proteins and complex mechanisms underlying the development of cotton S.E. remain largely unknown. In the present study, we performed a proteomics analysis and revealed that the differential expression of specific proteins involved in various biological processes was associated with cotton S.E. Among those proteins, several redox-related proteins were identified. The suppression of *GhAPXs* and *GhGSTL3* influenced cotton S.E. Data support the view that ROS homeostasis is crucial for initiating and maintaining dedifferentiation, while mild oxidative conditions promote redifferentiation, and there is an interplay between ROS and auxin homeostasis to modulate S.E. in cotton plants.

## EXPERIMENTAL PROCEDURES

### 

#### 

##### Plant Materials and Tissue Culture

The sterilized seeds of YZ1 (*Gossypium hirsutum L.*) were cultured on 1/2 MS (1/2 strength macro salts plus 15 g glucose, pH 6.0) at 28 °C in the dark for 7 days. The hypocotyls were excised from aseptic seedlings, dissected into ∼8 mm segments, and used as initial explants. The explants were subsequently cultured on MSB medium (MS medium plus B5 vitamins) containing 1.0 mg/l indol-3-ylbutyric acid (IBA) and 0.1 mg/l kinetin. After cultivation for 40 days, all explants were transferred to fresh MSB media for induction of embryogenic callus (EC) in which the medium contained twice the concentration of KNO_3_, with NH_4_NO_3_ free, and supplemented with 3% (w/v) glucose, 0.25% (w/v) Phytagel, 0.5 mg/l IBA, 0.15 mg/l kinetin, 1.0 g/l glutamine, and 0.5 g/l asparagine for embryo induction and maturation as previously described (6). Cultures were maintained at 28 ± 2 °C under a 14-h photoperiod (irradiance of 135 μmol/ms). As previously described (6), different time points/stages of explants (0 h, 2 d, 40 d, defined as nonembryogenic calluses, NECs), ECs and somatic embryos (globular embryos, GEs; torpedo embryos, TEs; and cotyledon embryos, CEs) were sampled and frozen at −70 °C until required for further analysis.

To generate *GhAPXs* and *GhGSTL3* suppression lines, gene-specific primers with attB1 and attB2 adaptors (listed in Table S1) were used, and the PCR products, respectively, cloned into pHellsgate4. RNAi vectors were introduced into *G. hirsutum* YZ1 plants by *Agrobacterium tumefaciens* using strains LBA4404. Two representative suppression lines for each of the genes *GhAPXs* and *GhGSTL3* were selected for further experiments. The culture conditions for the transgenic plants were conducted as described above.

##### Protein Extraction, 2-DE, and MALDI-TOF/TOF Analysis

For total protein extraction, a minorly modified procedure, based on our previous study (37), was used. The samples collected at different stages of cotton S.E. (0 h, 2 d, NECs, ECs, GE, TE, CE) were ground to a fine powder in liquid nitrogen. The powder was suspended in 30 ml of cold acetone containing 10% (w/v) trichloroacetic acid (TCA) and 1% (w/v) 2-mercaptoethanol for at least 15 min. After centrifuging at 9391 *g* (4 °C) for 15 min, the supernatant was carefully decanted, and the resulting pellet was washed twice in cold acetone containing 0.1% (w/v) dithiothreitol (DTT). The vacuum-dried powder was incubated in extraction buffer (30% sucrose, 100 mm Tris-HCl, pH 8.0, 2% sodium dodecyl sulfate (SDS), 2 mm phenylmethanesulfonyl fluoride (PMSF), 1% 2-mercaptoethanol, and an equal volume of Tris-saturated phenol, pH 8.0) for 30 min. The phenol phase was carefully collected and precipitated overnight with five volumes of 0.1 m ammonium acetate in methanol at −20 °C. The collected protein pellets were subsequently washed with 80% cold methanol, followed by washing with 100% cold methanol and acetone. After air drying, the pellets were dissolved in lysis buffer (7 m urea, 2 m thiourea, 4% 3–3-cholamidopropyl dimethylammonio-1-propanesulfonate (CHAPS), 1% DTT, and 2% v/v immobilized pH gradient buffer, pH 4–7). The protein concentration was determined using a 2-D quant kit (Bio-Rad).

Two-dimensional gel electrophoresis (2-DE) was performed according to the manufacturer's instructions (Bio-Rad). Total proteins (1.0 mg) from each sample were individually loaded onto immobilized pH gradient strips (17 cm, pH 4–7 nonlinear, Bio-Rad) with 300 μl rehydration buffer (7 m urea, 2 m thiourea, 4% CHAPS, 1% DTT, and 2% v/v immobilized pH gradient buffer, pH 4–7). The strips were rehydrated for 12 h at room temperature. Isoelectric focusing was performed using the following protocol: 50 V for 1 h, 500 V for 1 h, 1000 V for 1 h, and 10,000 V for 4 h, with a final step of 10,000 V for a total of 90 kVh. Each focused strip was equilibrated for 15 min with equilibration buffer (50 mm Tris-HCl, pH 8.8; 6 m urea; 30% glycerol; and 2% SDS; and 1% DTT), followed by a second equilibration step for an additional 15 min with equilibration buffer in which DTT was substituted with 2% (w/v) iodoacetamide. After equilibration, the immobilized pH gradient strips were fixed on top of vertical 12% acrylamide gels. Electrophoresis was performed using the Bio-Rad system (protean II XL) at 15 mA/gel for 1 h, followed by 45 mA/gel for 6 h until the bromphenol blue dye front reached the bottom of the gel.

The 2-D gels were stained with Coomassie Brilliant Blue solution. The stained gels were scanned using a GS-800 Calibrated Densitometer (Bio-Rad), and the protein spots were calculated using PDQuest software (Bio-Rad). After volumetric quantification and matching, spots of various intensities at different time points/stages of S.E. were analyzed using the Student's *t* test and calculated as a fold ratio with a threshold of *p* ≤ 0.05 and fold-change of ≥ 2 or ≤ 0.5. Three biological repeats were performed for each 2-DE image to find steadily repeatable DEP spots.

DEPs were excised from the gels, followed by sequential treatments, including destaining with 25 mm NH_4_HCO_3_ in 50% Acetonitrile (ACN) until the coomassie brilliant blue (CBB) disappeared, digestion with trypsin (Promega, Madison, WI) overnight at 37 °C, and extraction with extraction buffer (67% ACN and 5% TFA), as indicated by our previous study (37). The treated spots were then analyzed using an ABI 5800 MALDI-TOF/TOF Plus mass spectrometer (Applied Biosystems, Foster City, CA). Both the MS and MS/MS data were integrated using GPS Explorer V3.6 software (Applied Biosystems). Successfully identified proteins were characterized with a 95% or higher confidence interval using the MASCOT V2.3 search engine (Matrix Science, London, UK). The *Gossypium* expressed sequence tag (EST) database (release data 20120128; 2,476,590 sequences; 555,009,942 residues) was used for searching. The other search parameters were as our previous study (37), including the enzyme trypsin; partial modifications of cysteine carbamido methylation and methionine oxidization; fixed and variable modifications; one missed cleavage site; peptide tolerance of 100 ppm; and fragment mass tolerance of 0.5 Da.

##### Redox Disturbance and Auxin Effect on S.E. Process

To determine the effect of redox homeostasis on S.E., explants were cultured on MSB medium supplemented with diphenyleneiodium (DPI) at various concentrations (0, 1, 2, and 2.5 μm), or H_2_O_2_ (0, 25, 50 μm, 0.1, 0.5, and 1 mm), or 2 μm DPI + H_2_O_2_ (25 μm, 0.1 mm, and 0.5 mm). Explants were also cultured on MSB medium containing 0.1 mm buthionine sulfoximine (BSO), or 0.1 mm reduced glutathione (GSH), or 0.1 mm BSO + 0.1 mm GSH to investigate the effect of redox disturbance on dedifferentiation process. Besides, explants were also cultured on MSB medium containing ASA^1^ with different concentrations (0, 50, 100 μm) to detect whether ASA could complement the effect of *GhAPXs* suppression. The fresh weights of the explants were recorded at different time points/stages during S.E., and the proliferation rate was indicated as the increased fresh weight of the explants per gram. To investigate the effect of redox disturbance on the redifferentiation process, homogenized ECs were also treated with BSO and GSH, both at concentrations of 0.1 mm for 20 d, and the number of somatic embryos was counted.

To investigate the crosstalk between H_2_O_2_ and auxin, explants were cultured on MSB medium containing 2 μm DPI or 1 mm H_2_O_2_ supplemented with different concentrations of IBA (4.9, 14.7, 19.6, and 29.4 μm) or containing 5 μm 2,3,5-triiodobenzoic acid supplemented with different concentrations of H_2_O_2_ (25 μm and 100 μm). The proliferation rate was recorded as above.

##### ROS Detection and Measurement

To visualize ROS accumulation in BSO-treated cultures, explants in different stages of S.E. were incubated in 10 μm 2′,7′-dichlorofluorescein diacetate (2′, 7′-dichlorofluorescein diacetate) dissolved in dimethyl sulfoxide (DMSO) at a final concentration of 0.1% for 30 min in the dark at 30 °C, followed by washing twice with sterile water before imaging. Dichlorofluorescein fluorescence was imaged using stereo fluorescence microscopy.

For the quantification of H_2_O_2_ content, a H_2_O_2_ quantification kit (Sangon Biotech, Shanghai, China) was used. Samples (ca. 0.2 g fresh weight (FW)) were collected at different stages of S.E. development for the direct measurement of H_2_O_2_. The H_2_O_2_ was extracted as previously described (38). Samples were ground into powder in 1.6 ml precooled acetone, followed by shaking for 20 min and centrifuging at 4 °C (15,871 *g* for 15 min). The supernatant was added to a fresh centrifuge tube for further detection and H_2_O_2_ determination was performed according to the manufacturer's instructions.

##### ASA Measurement

ASA detection was performed according to Kampfenkel *et al.* with minor modifications (39). About 0.2 g samples were homogenized with 0.5 ml 5% (w/v) sulfosalicylic acid for 15 min on ice, and the mixture was then centrifuged at 11,000 *g* for 5 min at 4 °C. The supernatant was collected for further assay. Reactions were conducted following adding a mixture of 100 μl supernatant, 24 μl 1.84 m triethanolamine, 250 μl PBS (pH 7.4) containing 2.5 mm EDTA, and 10 mm DTT. The reactions were incubated in a water bath for 15 min at 25 °C. After that, 50 μl of 5% (w/v) N-ethylmaleimide were added to remove excess DTT. The chromogenic reaction was performed with addition of 200 μl 10% (w/v) TCA, 200 μl 44% (v/v) phosphoric acid, 200 μl 4% (w/v) a,a′-dipyridyl (dissolved in 70% ethanol), and 100 μl 3% (w/v) FeCl_3_. The sample was mixed and incubated in a water bath for 1 h at 42 °C. Absorbance was measured at 560 nm with an EnSpire® Multimode Plate Reader (PerkinElmer). Total ASA (ASA + DHA) was determined. The reduced ASA content determination was performed as the total ASA determination except DTT and N-ethylmaleimide were replaced by distilled water. Commercial ASA (Sigma-Aldrich) dissolved in double-distilled water was used for the calibration curve.

##### Auxin and Auxin Metabolites Extraction

Approximately 0.1–0.2 g of tissue from selected stages of S.E. were sampled and frozen at −70 °C until further analysis. The measurement of endogenous free IAA and IAA metabolites were performed as previously described (40), with some modifications. Approximately 200 mg of each sample were ground to a fine powder in liquid nitrogen, followed by extraction with 800 μl precooled 80% methanol solution containing 1% acetic acid, vigorous shaking in the dark overnight at 4 °C, and centrifugation at 15,871 *g* at 4 °C for 15 min. The supernatant was carefully collected, and the pellet was resuspended with additional 0.4 ml extraction buffer, extracted for 4 h at 4 °C, followed by centrifugation. The supernatants were combined and loaded onto an HLB column (Waters) and washed with 70% methanol solution containing 2% acetic acid. The filtrates were dried through evaporation under the flow of nitrogen gas and dissolved in 60 μl 10% methanol.

##### RNA Extraction, RT-PCR and qRT-PCR

Total RNA was isolated from selected samples as previously described (12). About 2 μg RNA were used for reverse transcription. The first-strand cDNAs were synthesized with SuperScript III reverse transcriptase (Invitrogen, Carlsbad, CA) and used as templates. qRT-PCR was performed using the ABI Prism 7000 system (Applied Biosystems, Foster City, CA), and the expression levels of selected genes were normalized to *GhUB7* (GenBank accession number: DQ116441) using the 2^-ΔCt^ calculated method as previously described (41). qRT-PCR was conducted with three biological replicates and three technical replicates for each biological replicate. The primers for qRT-PCR and RT-PCR are listed in Tables S1 and S2, respectively. All primers were designed using Primer Premier software version 5.0.

##### Statistical Analysis

All graphical data were generated from three biological replicates, and the values are presented as the means ± S.D. Statistical significance was determined using one-way ANOVA analysis, and *p* values < 0.05 were considered statistically significant.

## RESULTS

### 

#### 

##### Proteomics Analysis and Identification of DEPs During Cotton S.E

**Fig. 1.**
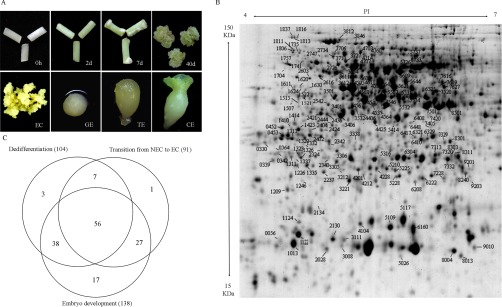
**Morphological characteristics of different time-points/stages and 2-DE map of differentially expressed proteins (DEPs) during cotton somatic embryogenesis (S.E.)**. (*A*) Different stages of cotton S.E. 0 h: initial hypocotyl explants used as controls; 2 d: explants cultured for 2 d; 40 d: explants cultured for 40 d, representing nonembryogenic callus (NEC); ECs: embryogenic calluses; GE: globular embryo; TE: torpedo embryo; CE: cotyledon embryo. (*B*) Identification of DEPs during S.E. on the 2-DE map. (*C*) Venn diagram of DEPs identified in each of three different developmental stages of cotton S.E.

S.E. initiation and development in cotton plants is followed by sequential morphological and dynamic changes. No visible morphological changes were observed on the explants cultured for 2 d (Fig. 1*A*), and cell expansion was observed through histological observation as previously described (6). After culturing for 7 d, both ends of the explants expanded and formed callus. Subsequently, mass calluses proliferated and developed into ECs, followed by differentiation into GEs, TEs, and CEs (Fig. 1*A*).

Based on the morphological events, the samples from 0 h, 2 d, NEC, EC, GE, TE, and CE were collected for comparative proteomics. The numbers of independent protein spots detected varied for different developmental stages, ranging from 838 to 1360 spots observed on 2-D gels. A total of 155 significantly expressed DEPs were successfully identified during S.E. using MALDI-TOF/TOF and the parameters described above (F ≥ 2 or ≤ 0.5 and *p* ≤ 0.05) (Fig. 1*B*, Fig. S1). After removing the redundant proteins, 149 unique proteins were identified (Fig. 1*C*). These identified DEPs were categorized according to functional categories as indicated by molecular function (Table S3). A large proportion of these proteins have binding ability, such as general regulatory factors (14-3-3 proteins), glutamine synthetase, ATP synthase, and translation elongation factors. Besides, a large amount of the DEPs possess oxidoreductase activity, including several ROS-related proteins as well as HSP70 proteins and ribulose-bisphosphate carboxylases. DEPs have transferase activity, such as glutathione S-transferase family proteins and sedoheptulose-bisphosphatase, were also characterized. And DEPs confer hydrolase activity and isomerase activity and several unknown proteins were also identified (Table S3). Further detailed information for these proteins was annotated and described in Table S3.

**Fig. 2.**
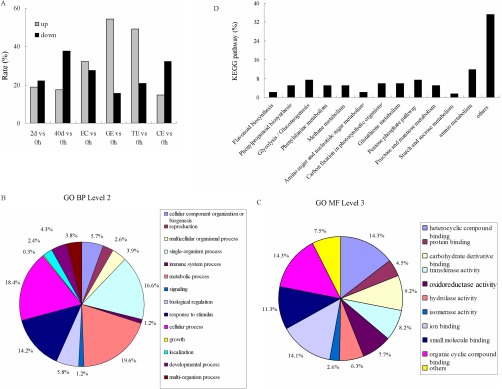
**Histogram and functional categories of differentially expressed proteins during S.E.** (*A*) Percentage of proteins up/down-regulated at different time points during S.E. (*B*, *C*) Functional categories of differentially expressed proteins assigned with GO term at level two biological processes (*B*) and level 3 molecular functions (*C*). The percentages were calculated in relation to all differentially expressed proteins in S.E. (*D*) Percentages of proteins involved in various pathways based on Kyoto Encyclopedia of Genes and Genomes analysis.

Spatial analysis was conducted on DEPs to confirm the degree of overlap during dedifferentiation, the transition from NEC to EC, and somatic embryo development during cotton S.E. as previously described (6). Approximately 104, 91, and 138 differentially expressed proteins were identified, representing the three different developmental stages (Fig. 1*C*). Among these, 37.6% (56) of DEPs were detected during all three developmental stages, of which 2.01% (3) were modulated during dedifferentiation, 0.67% (1) during the transition from NEC to EC, and 11.4% (17) during the somatic embryo development (Fig. 1*C*). Among the 149 identified proteins, 18.8% (28) were up-regulated (compared with 0 h hypocotyls) at 2 d, 17.4% (26) at NEC, 32.2% (48) at EC, 54.4% (81) at GE, 49.0% (73) at TE, and 14.8% (22) up-regulated at CE. 22.1% (33) were down-regulated at 2d, 37.6% (56) at NEC, 27.5% (41) at EC, 15.4% (23) at GE, 20.8% (31) at TE, and 32.2% (48) down-regulated at CE (Fig. 2*A*).

Gene ontology analysis was performed using Blast2GO. Under the level 2 biological process category, cellular and metabolic processes were most commonly represented, accounting for nearly one-quarter of all proteins (Fig. 2*B*), followed by the “single-organism process” (16.6%) and “response to stimulus” (14.2%). Other important biological processes, such as development (4.3%), reproduction (2.6%), and growth (0.3%) were also identified (Fig. 2*B*). Based on the molecular function category at level 3, a number of the proteins were assigned as having binding properties and a category of proteins possessing oxidoreductase activity were also well represented (Fig. 2*C*).

Analyses using Kyoto Encyclopedia of Genes and Genomes were conducted to distinguish the biological pathways involved in cotton S.E. Among these pathways, total amino metabolisms accounted for 11.76%, followed by the pentose phosphate pathway (7.35%) and the glycolysis/gluconeogenesis pathway (7.35%). Glutathione metabolism (5.88%), phenylpropanoid biosynthesis (5.15%), and phenylalanine metabolism (5.15%) were also well represented (Fig. 2*D*).

**Fig. 3.**
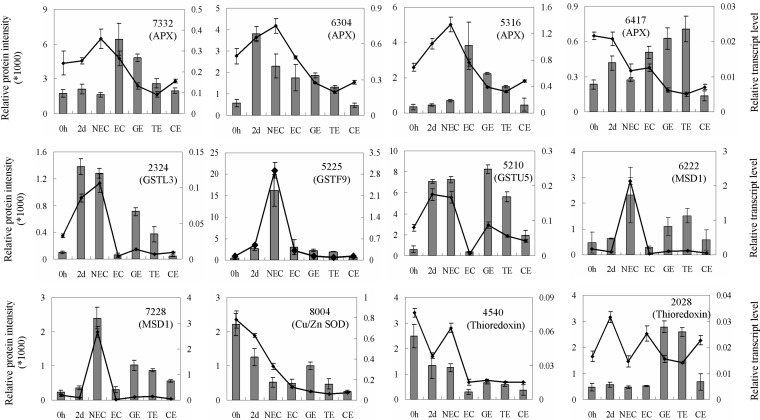
**Comparison of the expression profiles between protein and transcript levels for several redox-related proteins.** The quantification of the protein intensities and corresponding relative transcript levels are represented as bar and line charts, respectively. qRT-PCR was performed to determine the transcript levels of the selected proteins. The expression levels were normalized to *GhUB7*, and the relative expression was calculated as 2^-ΔCT^, ΔCT = (C_T, Target_–C_T, GhUB7_)_Time x_. The error bars represent ± S.D. of three biological replicates. APX, four DEP spots (SSP7332, 6304, 5316, 6417) were identified as ascorbate peroxidases; GSTL3, DEP spot 2324 was identified as glutathione transferase L3; GSTF9, DEP spot 5225 was identified as glutathione transferase Phi 9; GSTU5, DEP spot 5210 was identified as glutathione transferase Tau 5; MSD1, two specifically identified protein spots 6222 and 7228 were identified as MnSOD; Cu/Zn SOD, DEP spot 8004 was identified as Cu/Zn SOD; Thioredoxin, two DEP spots 4540 and 2028 were identified as thioredoxins.

Plants possess a series of ROS scavenging enzymes for protection against ROS damage (15), and it is interesting to note that protein levels did not always correlate with mRNA abundance (42). As shown in Fig. 3, the mRNA expression levels of the three cytoplasmic APXs (SSP7332, 6304, 5316) were consistent with the protein levels, while the transcript level of stromal ascorbate peroxidase (SSP6417) showed an opposite expression pattern compared with the protein level (Fig. 3). The transcript levels for three identified GST proteins were also consistent with the expression patterns at the protein level (Fig. 3), and the transcript levels of the identified SOD enzymes (SSP6222, 7228, 8004) were consistent with the protein expression profiles observed during S.E. (Fig. 3). Similarly, the transcript profile of thioredoxin (SSP4540) was also consistent with the protein profile, but the transcript level of a second thioredoxin (SSP2028) showed an opposite protein expression pattern (Fig. 3). The results indicated that these ROS-related proteins might be involved in cotton S.E.

Except for ROS-related proteins, the expressions of DEPs involved in amino acid metabolisms, such as diaminopimelate epimerase (SSP3401), glutamine synthetase 1 (SSP4546), spermidine synthase 2 (SSP1521), arginosuccinate synthase (SSP6609), 3-isopropylmalate dehydratase (SSP3212), ketol-acid reductoisomerase (SSP4760), and S-adenosylmethionine synthetase (SSP5644, SSP5414), were detected in the transcript level and the protein level. However, the expression profiles of these genes on transcript level were not correlated with the protein level (Fig. S2). The expression profiles of aldolase-type TIM barrel family protein (SSP1620, SSP1624, SSP1630) and pfkB-like carbohydrate kinase family protein (SSP2421, SSP2436, SSP3406), which assigned to the pentose phosphate pathway, were also investigated in both transcript level and protein level. The mRNA expression levels of genes involved in the pentose phosphate pathway were also not consistent with the protein levels, except for SSP2436 and SSP3406, whose expression profiles were consistent in transcript level and protein level (Fig. S2).

Among the DEPs, proteins involved in flavonoid biosynthesis such as chalcone synthase (SSP7619) and chalcone isomerase (SSP1226) were also characterized (Fig. S3*A*). The mRNA expression levels of chalcone synthase (CHS) (SSP7619) and chalcone isomerase (CHI) (SSP1226) were consistent with the protein levels (Fig. S3*B*). As well, the transcript profiles of other main genes involved in flavonoid biosynthesis were detected. The expression patterns of all these flavonoid genes were nearly similar during S.E. processes (Fig. S3*B*). The endogenous contents of flavonoids during SE processes were also consistent with the genes expression profile. The endogenous flavonoids were abundantly accumulated in 2 d but were less during subsequently time points/stages (Fig. S3*C*).

##### Defective Cytoplasmic GhAPXs-Mediated ROS Accumulation Regulates Cotton S.E

**Fig. 4.**
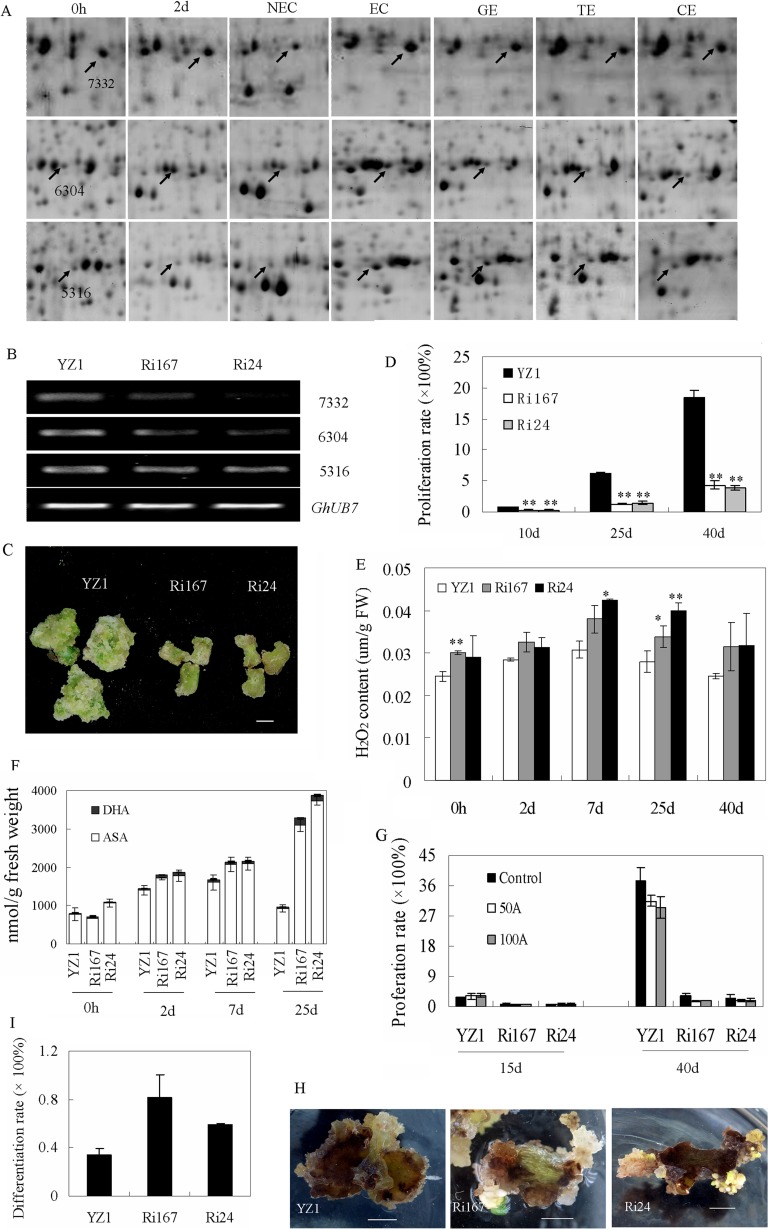
**Reduced expression of *GhAPXs* inhibits dedifferentiation but accelerates redifferentiation.** (*A*) Differentially expressed GhAPX proteins (SSP7332, 6304, 5316) shown on 2-DE maps during S.E. (*B*) The expression of three identified APX proteins (SSP7332, 6304, and 5316) were down-regulated in the *GhAPX* interference lines. (*C, D*) Dedifferentiation was significantly retarded in suppression lines. The growth rates were recorded at different time-points/stages during S.E. (*E*) H_2_O_2_ content was significantly higher in interference lines relative to wild-type plants during S.E. development. (*F*) The content of endogenous ASA metabolism was altered by *GhAPXs* suppression. ASA, reduced ascorbic acid; DHA, oxidized ascorbic acid. (*G*) The retarded dedifferentiation process caused by *GhAPXs* suppression was not complemented by exogenous application of reduced ASA. (*H*, *I*) Redifferentiation was accelerated in the suppression lines. The images were captured after culturing for 40 d (*C*) and 120 d (*H*). The differentiation rate was recorded after culturing for 120 d. All bars represent 0.5 cm. YZ1 was used as a control. Ri167 and Ri24 represent the two suppression lines.

APXs play pivotal roles in maintaining intracellular ROS homeostasis (30). Considering the differential expression of APXs during S.E. (Fig. 4*A*), a disruption of these *GhAPXs* was conducted in cotton to clarify the function of ROS scavenging enzymes on S.E. Two representative interference lines (Ri167 and Ri24) were obtained. The expression of the three identified cytoplasmic *GhAPXs* (SSP7332, 6304, 5316) was down-regulated in the two interference lines (Fig. 4*B*), and S.E. initiation and dedifferentiation were significantly inhibited in these lines compared with wild type (Fig. 4*C*). The cell proliferation rates of interference lines were sharply decreased during dedifferentiation (Fig. 4*D*), accompanied by the higher endogenous H_2_O_2_ content in the down-regulated lines than in wild-type plants during S.E. (Fig. 4*E*). The contents of reduced ASA were elevated in *GhAPXs* interference lines as compared with wild type during the dedifferentiation process. As well, the contents of total ASA (ASA+DHA) were altered in *GhAPXs* suppression lines (Fig. 4*F*). The effect of exogenous application of ASA on dedifferentiation process was also detected in both *GhAPXs* interference lines and wild type. There were no significant differences between ASA treatment and control on the cell proliferation rate of both *GhAPXs* interference lines and wild type after culturing for 15 d (Fig. 4*G*). As culturing for 40 d, the cell proliferation rate was retarded by ASA treatment both in *GhAPXs* interference lines and wild type (Fig. 4*G*), which were consistent with the disturbance of ASA homeostasis in *GhAPXs* interference lines. Furthermore, ECs and somatic embryos were observed earlier in *GhAPXs* interference lines than in wild type (Fig. 4*H*). After culturing for 120 d, the differentiation rates were 81.9% and 59.2% in down-regulated lines compared with 34.4% in wild-type controls (Fig. 4*I*). These results suggested that GhAPXs play important roles during S.E., and the alteration of S.E. process through APX suppression might be associated with the elevated endogenous H_2_O_2_ content.

##### Suppressing the Expression of GhGSTL3 Promotes the Redifferentiation during Cotton S.E

**Fig. 5.**
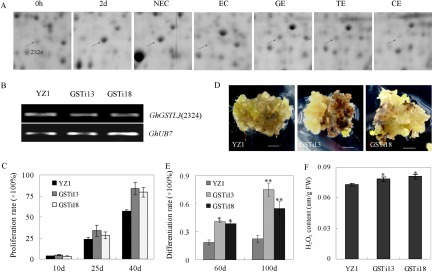
**Suppressing the expression of *GhGSTL3* promotes the redifferentiation during cotton S.E.** (*A*) GhGSTL3 proteins (SSP2324) were identified in 2-DE. (*B*) PCR shows the expression of *GhGSTL3* was down-regulated in *GhGSTL3* suppression lines. (*C*) The dedifferentiation was slightly affected in *GhGSTL3* interference lines. (*D*, *E*) *GhGSTL3* negatively regulates redifferentiation. The differentiation rates were recorded after culturing for 60 and 100 d. The images were captured after culturing for 100 d. All bars represent 0.5 cm. (*F*) The H_2_O_2_ content was higher in *GhGSTL3* suppression lines after culturing for 50 d. YZ1 was used as the wild-type control, and GSTi13 and GSTi18 represent the two suppression lines.

Other than APXs, several GST-related proteins were also identified as differentially abundant throughout embryogenesis (Fig. 5*A*, Table S3). In the present study, *GhGSTL3* (SSP2324) interference was generated in cotton plants and two representative lines were selected (Fig. 5*B*). Dedifferentiation was marginally altered in *GhGSTL3* interference lines relative to wild-type plants. There were no apparent distinction between interference lines and wild-type plants at the preliminary stage of dedifferentiation. However, callus production was greater in interference lines than in wild-type tissues cultured for 40 d (Fig. 5*C*). As the culture period progressed, the transition from NEC to EC was obviously accelerated, and somatic embryos were observed in *GhGSTL3* suppression lines compared with wild-type plants (Fig. 5*D*). The differentiation rates were, respectively, 40.8% and 38.8% in interference lines and 18.5% in wild-type plants, and 75 and 55% in interference lines but only 22.3% in wild-type plants after culturing for 60 d and 100 d, respectively (Fig. 5*E*). The endogenous H_2_O_2_ contents were significantly higher in interference lines compared with wild type (Fig. 5*F*).

##### ROS Homeostasis Is Crucial for Cotton S.E

**Fig. 6.**
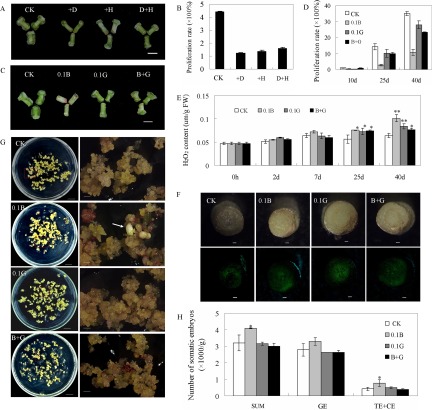
**Effects of ROS perturbation on the dedifferentiation and redifferentiation of cotton S.E.** (*A*, *B*) Dedifferentiation, measured as cell proliferation, was inhibited by both DPI and H_2_O_2_ treatment, and the inhibition effect was partially reversed by treatment with DPI + H_2_O_2_. (*C*, *D*) Dedifferentiation was inhibited by both BSO and GSH treatment. GSH could partially rescue the inhibitory effect of BSO. (*E*) The endogenous H_2_O_2_ content of samples cultured on different BSO and GSH treatments. (*F*) ROS detection through 2′, 7′-dichlorofluorescein diacetate staining after culturing for 2 d. (*G*, *H*) BSO treatment promoted the transition of EC to somatic embryos. The number of total somatic embryos and globular embryos was counted after ECs were treated for 20 d. CK: explants (*A–F*) and ECs (*G*, *H*) cultured on normal MSB medium; +D: explants cultured on medium containing 2 μm DPI; +H: explants cultured on medium containing 1 mm H_2_O_2_; D+H: explants cultured on medium containing 2 μm DPI and 25 μm H_2_O_2_; 0.1B: explants (*C*, *D*) and ECs (*F*, *G*) cultured on medium supplement with 0.1 μm BSO; 0.1G: explants (*C*, *D*) and ECs (*F*, *G*) cultured on medium supplement with 0.1 μm GSH; B+G: explants (*C*, *D*) and ECs (*F*, *G*) cultured on medium supplement with 0.1 μm BSO plus 0.1 μm GSH. The images were captured after culturing for 15 (*A*, *C*) and 20 d (*G*). The bars represent 0.5 cm (*A*, *C*), 0.25 mm (*F*), 1 cm (*G, left*) and 1 mm (*G, right*).

To further investigate whether ROS signaling is involved in cotton S.E., explants were cultured on medium supplemented with different concentrations of DPI, a widely used inhibitor of NADPH oxidase, a ROS-generating enzyme (43). The dedifferentiation process was significantly retarded with DPI treatment, more severe as the concentration increased (Figs. 6*A* and 6*B* and Fig. S4*A*), indicating that ROS was necessary for dedifferentiation during cotton S.E. Similar results were obtained after H_2_O_2_ treatments (Figs. 6*A* and 6*B*). The initiation and dedifferentiation process in cotton plants during S.E. was seriously retarded with increasing H_2_O_2_ concentration (Figs. S4*B* and S4*D*). Consistent with these results, the dedifferentiation and cell proliferation rates were partially rescued in the presence of DPI plus appropriate concentrations of H_2_O_2_ (Figs. 6*A* and 6*B*, Figs. S4*C* and S4*D*).

GSH and BSO (specific inhibitors of GSH biosynthesis) were used to mimic ROS perturbation *in vivo*. Explants were cultured on medium containing 0.1 mm BSO (Figs. 6*C* and 6*D*). Consistent with H_2_O_2_ treatments, the initiation and dedifferentiation of explants cultured on medium containing BSO were nearly inhibited (Figs. 6*C* and 6*D*). With GSH treatment, the dedifferentiation process was slightly retarded (Figs. 6*C* and 6*D*). To modify the effect of BSO on cotton S.E., GSH was applied to medium containing 0.1 mm BSO. The negative effect of BSO on S.E. was partially restored by GSH (Figs. 6*C* and 6*D*). These results suggest that ROS is a key factor for dedifferentiation and ROS homeostasis is critical for initiating dedifferentiation.

Endogenous levels of H_2_O_2_ in BSO- or GSH-treated samples were determined during cotton S.E. There were no significant differences found between treatments and control conditions at the preliminary stage of cotton S.E.; however, the H_2_O_2_ content was significantly higher in BSO-treated samples compared with samples cultured under normal conditions during the late stages of dedifferentiation (Fig. 6*E*). The H_2_O_2_ level in explants cultured on medium containing GSH was slightly elevated in 2 d cultures. Thereafter, no significant changes were observed until 40 d after culturing (Fig. 6*E*), consistent with the phenotype observed. The increased H_2_O_2_ content in response to BSO treatment was reduced after applying 0.1 mm GSH to medium containing 0.1 mm BSO (Fig. 6*E*).

The ROS accumulation during the development of cotton S.E. was also investigated under both control and treated conditions, using 2′, 7′-dichlorofluorescein diacetate staining. After culturing for 2 d, the fluorescence intensity was significantly elevated under both BSO and GSH treatment conditions compared with control (Fig. 6*F*). During S.E., BSO treatment induced stronger fluorescence intensity compared with controls, while no significant differences were observed between GSH treatment and the controls (Fig. S5). These results indicated that inhibition of GSH activity by BSO treatment altered the redox state and elevated ROS accumulation *in vivo*.

The differentiation process was also examined by BSO and GSH treatments. With embryogenic callus cultured on medium containing 0.1 mm BSO for 20 d, the number of total somatic embryos was higher compared with the control (Figs. 6*G* and 6*H*). No significant difference was observed in the number of globular embryos following BSO treatment compared with controls, but significantly more torpedo and cotyledon embryos were observed with BSO treatment (Figs. 6*G* and 6*H*). For GSH treatment, no significant differences in the number of total somatic embryos, and the different development stages of somatic embryos were observed compared with control (Figs. 6*G* and 6*H*). The accelerated differentiation through BSO application was modified through additional GSH supplement (Figs. 6*G* and 6*H*). These results suggest that an oxidative environment promotes differentiation and accelerates somatic embryo development.

##### ROS Perturbation Negatively Impacts Auxin Homeostasis during Cotton S.E

**Fig. 7.**
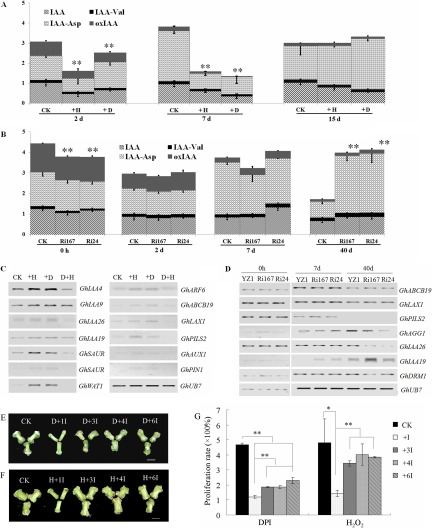
**ROS perturbation negatively impacts auxin homeostasis during cotton S.E.** (*A*) The contents of free IAA, IAA-Asp, IAA-Val, and oxIAA were determined in cultures treated with DPI and H_2_O_2_ at the early stage of cotton S.E. (*B*) The contents of free IAA, IAA-Asp, IAA-Val, and oxIAA were analyzed in GhAPX suppression lines and wild-type controls. (*C*) The expression of auxin-related genes was altered after DPI or H_2_O_2_ treatment. (*D*) The expression of auxin-related genes was altered in suppression lines relative to wild type. (*E–G*) The inhibition effect caused by DPI (*E*) or H_2_O_2_ (*F*) on dedifferentiation was partially rescued through the application of auxin. CK: samples cultured on control medium; +H: samples cultured on medium containing 1 mm H_2_O_2_; +D: samples cultured on medium containing 2.5 μm DPI (*A*) and 2 μm DPI (*C*); 2D+1I/3I/4I/6I: explants cultured on medium supplement with 2 μm DPI and 4.9, 14.7, 19.6, and 29.4 μm IBA, respectively, compared with the normal control; 1H+1I/3I/4I/6I: explants cultured on medium supplement with 1 mm H_2_O_2_ and 4.9, 14.7, 19.6, and 29.4 μm IBA, respectively. The images were captured, and the growth rates were recorded after culturing for 15 d. The bars represent 0.5 cm.

Transcriptional analysis revealed that ROS can influence plant development by modulating auxin-dependent gene expression in *Arabidopsis* (30–33). To examine the interaction between ROS and auxin signaling in cotton S.E., the contents of free IAA and IAA metabolites were analyzed in cultures treated with DPI or H_2_O_2_ during different stages of S.E. The contents of free IAA, IAA-Asp, IAA-Val, and oxIAA were significantly reduced in both DPI- and H_2_O_2_-treated cultures compared with untreated controls after 2 d and 7 d culturing (Fig. 7*A*). After culturing for 15 d, the content of free IAA was decreased in DPI- and H_2_O_2_-treated cultures, but there was no significant differences found in the content of IAA-Asp, IAA-Val, and oxIAA between the treatments and control at that stage (Fig. 7*A*). Free IAA and IAA metabolite contents were also examined in *GhAPXs-*defective transgenic lines during S.E. The metabolic profile and free IAA content were comparable between interference lines and wild-type plants during S.E., with a slight accumulation for explants at 40 d. No significant differences in oxIAA content were observed between *GhAPXs*-defective transgenic plants and wild-type plants (Fig. 7*B*). However, the IAA-Asp content was lower in suppression lines when cultured for 7 d but higher in suppression lines when cultured for 40 d compared with wild-type plants. There were no obvious differences in IAA-Val content with wild-type plants at different time points/stages, but the IAA-Val content was continuously elevated in suppression lines (Fig. 7*B*), indicating the metabolism patterns of IAA-Asp and IAA-Val was dependent on *GhAPX* expression during S.E.

Transcription analysis was carried out to elucidate the effect of the disruption of ROS homeostasis on the expression of genes involved in auxin transport and signaling (*AUX/IAAs*, *SAUR*-like genes, *GhABCB19*, *GhLAX1*, *GhPILS2*, *GhAUX1,* and *GhPIN1*). Expression levels of these genes increased after both H_2_O_2_ and DPI treatment (Fig. 7*C*). The transcription levels of several auxin transport and response genes were also altered in *GhAPXs* defective transgenic lines compared with wild-type plants at different time points/stages during cotton S.E. At 0 h, no obvious differences were observed between down-regulated lines and wild-type plants. However, the expression of these genes was differentially altered during S.E. development. By 7 d of culture, expression of *GhPILS2*, *GhAGG1,* and *GhIAA19* was relatively increased in suppression lines, though the other genes showed no obvious changes (Fig. 7*D*). By 40 d of culture, expression of the auxin transport genes *GhABCB19* and *GhLAX1* was increased in the suppression lines compared with wild-type tissues, while expressions of the auxin response genes *GhAGG1*, *GhIAA26,* and *GhDRM1* were reduced in down-regulated lines compared with wild-type plants, while *GhIAA19* expression was relatively increased (Fig. 7*D*). These results revealed that auxin signaling and transport were altered in *GhAPXs* interference lines.

Consistent with S.E. inhibition through DPI or H_2_O_2_ treatment, the recovery of growth could be observed by application of auxin to the medium containing 2 μm DPI (Fig. 7*E*). A similar restoration was also observed with the addition of auxin to medium supplemented with H_2_O_2_ (Fig. 7*F*). The degree of restoration was correlated with the auxin concentration, increasing as the level of auxin application increased under both DPI and H_2_O_2_ treatment (Fig. 7*G*).

## DISCUSSION

Early plant development is characterized by changes in the levels of key cellular proteins, as revealed by proteomics studies (8, 42). S.E. is considered as both an efficient pathway for plant propagation and a feasible model for investigating the early regulatory and morphogenetic events in plant embryogenesis (1, 3). Transcriptome analysis of cotton S.E. have been previously described (6, 12). Given the potentially variable correlation between mRNA and protein levels for individual genes (42), proteomics studies are essential to understand the regulatory mechanisms underlying cotton S.E. During dedifferentiation and redifferentiation processes of cotton S.E., 5076 differential expressed genes (DEGs) were identified through transcriptome analysis (6). There were 67 common DEGs identified both by proteomics and transcriptome analysis (Fig. S6*A*). Gene Ontology (GO) analysis indicated that the commonly identified genes were mostly involved in apoplast, ROS metabolic process, glycolytic/gluconeogenesis process, and hexose biosynthetic process (Fig. S6*B*). Notably, several of these common genes were also participated in glutathione metabolism as indicated by Kyoto Encyclopedia of Genes and Genomes analysis (Fig. S6*C*). Both the two approaches clarified that ROS-related genes were differentially expressed during cotton S.E. process, indicating that ROS might be involved in cotton S.E. process.

Ascorbate peroxidases (APXs) play important roles in ROS scavenging and are involved in many development programs (30, 44). Changes in endogenous ascorbate redox status have an effect on somatic embryo development and an oxidized environment benefit somatic embryo maturation (45–47). In the present study, several differentially expressed GhAPXs were identified (Fig. 3, Fig. 4*A*). The transgenic suppression of three identified APX proteins (SSP7332, 6304, 5316) changed endogenous ascorbate metabolism and induced endogenous H_2_O_2_ accumulation (Figs. 4*E*, 4*F*). The dedifferentiation process on culturing tissues for S.E. induction was retarded, while redifferentiation was promoted in GhAPXs suppression lines compared with wild-type tissues (Fig. 4). This suggests the involvement of ROS-scavenging enzymes in cotton S.E. Plant GSTs also act as ROS-scavenging enzymes associated with responses to stresses, hormone signaling, and developmental changes (48–50). The suppression of GhGSTL3, which was identified as being differentially expressed during S.E., had no significant effect on dedifferentiation, but redifferentiation was accelerated, and increased H_2_O_2_ levels were observed compared with wild-type tissues (Fig. 5). The elevated growth rate of GhGSTL3 suppression lines during the late stage of dedifferentiation might reflect the accelerated redifferentiation in GhGSTL3 suppression lines (Figs. 5*D* and 5*E*), as EC and NEC showed contrasting density (34).

The changing abundances of ROS-scavenging enzymes during S.E. suggests that ROS signaling might be important for regulating S.E. development. Dedifferentiation and cell proliferation were consistently retarded following treatment with DPI (Fig. 6*A*, Fig. S4*A*), which is an inhibitor of flavoenzymes, particularly NAD(P)H oxidase. NADPH oxidase plays a vital role in root development through the generation of ROS, which regulates cell expansion through the activation of Ca^2+^ channels (51). The inhibition of ROS production by blocking the activity of NADPH oxidase using DPI can phenocopy the *rhd2* mutant defective in NADPH oxidase (51). Localized ROS production is detectable at the pollen tube apex, and the inhibition of pollen tube elongation was observed after treatment with DPI or the suppression of endogenous NADPH oxidase expression (52). This suggests that NADPH oxidase-generated ROS and ROS signaling are critical for various plant developmental processes. The application of H_2_O_2_ to cotton tissues also treated with DPI was able to partially rescue this ROS inhibition and cell proliferation (Fig. 6*A*), further confirming that ROS is necessary for the initiation and development of tissue dedifferentiation.

Redox regulation is an elaborate mechanism utilized by plants to perceive and respond to perturbations in the ROS concentration (16). ROS can serve as signaling molecules involved in diverse metabolic processes, but at high concentrations, ROS can induce cell cycle arrest and apoptosis (15, 25). Also, ROS could cause oxidative modifications of proteins and affect their functionality, arising from modification of a wide range of amino acids (53–55). Protein carbonylation was regarded as a marker of protein oxidation and such posttranslational modification can result in the loss of function of target proteins (53, 56). Protein carbonylation has shown to be target specific and targets enzymes of photosynthesis and energy, as well as amino acid metabolism and other proteins, including stress proteins, HSP70 chaperones, and translation elongation factors (54, 56). Protein carbonylation plays roles in metabolic control and acts as a signal in physiological transitions in plants (56). The dose-dependent retardation of dedifferentiation in explants cultured on medium containing different levels of H_2_O_2_ revealed that excess ROS negatively impacts dedifferentiation (Fig. 6, Fig. S4). The application of BSO effectively decreased the biosynthesis of GSH, a key antioxidant in ROS scavenging and redox homeostasis maintenance, leading to a disturbance of the endogenous redox balance (57). Consistent with the negative effect of excess H_2_O_2_ on dedifferentiation, the initiation and development of dedifferentiation were largely blocked by BSO treatment (Fig. 6*C*). Although the H_2_O_2_ content in BSO-treated explants was comparable with that in control plants during the early stage of dedifferentiation (Fig. 6*E*), endogenous ROS levels became elevated during BSO treatment, as indicated through 2′, 7′-dichlorofluorescein diacetate staining (Fig. 6*F*, Fig. S5). In wheat, the inhibition of callus regeneration and overall efficiency of transformation were also observed after BSO treatment or the silencing of either of the GSH biosynthesis genes *GSH1* and *GSH2* (58). Our results suggested that redox status plays important roles in regulating dedifferention. As well, several homologues of the targets for posttranslational modifications were characterized in our identified DEPs, such as HSP70, ribulose-bisphosphate carboxylases, rubisco activase, ATP synthase, 14–3-3 proteins, glutamine synthetase, glutathione S-transferases (GSTs), and sedoheptulose-bisphosphatase (Table S3). Therefore, we speculated that posttranslational modifications of target proteins might be involved in S.E. metabolic control.

Consistent with these observations, the addition of GSH to BSO-containing medium could partially reverse the inhibitory effect of BSO on S.E. (Figs. 6*C* and 6*D*). Unexpectedly, the application of GSH slightly increased the H_2_O_2_ content during dedifferentiation and slightly suppressed the dedifferentiation process (Figs. 6*C* and 6*D*). It has been reported that GSH is recruited into the nucleus during cell proliferation, and the sequestration of GSH in the nucleus is accompanied by H_2_O_2_ accumulation (59). Thus, we speculate that the exogenous application of GSH might alter the endogenous GSH pool in the cytoplasm and nucleus, thereby influencing the normal cell proliferation cycle.

The roles of ROS as important regulators in plant development are potentially as diverse as hormonal signal transduction (14, 15). An oxidative environment plays a positive role in somatic embryo development in plants through enhanced cell division (18). Consistent with this, the transgenic suppression of *GhAPXs* increased the endogenous H_2_O_2_ content and accelerated the redifferentiation process (Figs. 4*E*–4*G*). Similar results were observed through the suppression of *GhGSTL3* (Figs. 5*D*–5*F*). The application of BSO has also been found to increase white spruce somatic embryo yield and quality, and these effects could also be phenocopied through alterations in the glutathione redox state through the experimental manipulation of endogenous reduced (GSH) and oxidized (GSSG) glutathione levels (57, 60). Similarly, BSO treatment positively affected cotton somatic embryo formation and development (Figs. 6*G* and 6*H*).

Stress responses were involved in S.E. process as demonstrated by previous work (12). N compounds, particularly glutamine, were important for the proliferation and maturation of somatic embryos in various species (61, 62). Enhanced cell proliferation and inhibited differentiation were observed as ammonium supply was blocked, which were reversed by resupply of glutamine as the form of nitrogen source, because nitrogen supply intrigued oxidative stress response (61). Therefore, a balanced nitrogen supply and metabolism might be critical for plant embryogenesis. Glutamine synthetases play specific roles in nitrogen metabolism during embryogenesis (63). In our work, two glutamine synthetase proteins were identified, which were involved in amino acid metabolism. We speculated that amino acid metabolism might be involved in S.E. process through modulating nitrogen metabolism, which correlated with oxidative stress response. Glutamine synthetase was a target for carbonylation; we also wonder whether enzymes in amino acid metabolism were referred to posttranslational modifications and then to play roles in metabolic control.

Proline synthesis from glutamate plays an important role in modulating plant cellular redox potential under stress conditions (64). Enhanced proline synthesis was considered to improve oxidative pentose phosphate pathway activity. Oxidative pentose phosphate pathway activity activation is traditionally correlated with high rates of cell division and differentiation (64). The activation of proline-linked pentose phosphate pathway plays a positive role in embryo formation that might be linked with endogenous cytokinin, auxin, and phenolic biosynthesis required for somatic embryogenesis (65). As we indicated, redox signaling was involved in SE process and proteins participating in the pentose phosphate pathway were identified during SE process. There might be a similar mechanism involved in cotton somatic embryogenesis. Carbonylation of sedoheptulose-bisphosphatase leads to enzyme inactivation, thus inhibiting carbon assimilation efficiency, resulting in growth and development retardation (66). Sedoheptulose-bisphosphatase was a critical enzyme in the pentose phosphate pathway, and differentially identified in our work, but its links to S.E. needs further experiments.

Flavonoids are polyphenolic compounds and have great potential to inhibit the generation of ROS and scavenge excess ROS once they are formed (67). Flavonoids are also natural inhibitors of auxin transport (68). Silencing the flavonoid pathway inhibits root nodule formation in *Medicago truncatula* due to blocked auxin transport (69). Flavonoids modulate the activity of auxin-transporting p-glycoproteins and likely modulate regulatory proteins activity (68). Flavonoid compounds participate in auxin transport arising from scavenging of ROS (68). Both the expression of genes in flavonoid biosynthesis and endogenous flavonoid compounds were significantly accumulated at the initiation stage of SE and might play roles in scavenging ROS and modulate auxin transport.

There is now good evidence to show that ROS signaling interacts with hormone networks to integrate extrinsic signals into developmental programs and stress tolerance responses (20). Auxin is important for both zygotic embryogenesis and S.E. induction, and development and auxin responses are concentration dependent (6, 21). Optimum endogenous auxin levels should be rigorously controlled through auxin homeostasis-maintaining mechanisms, including biosynthesis, conjugate formation, degradation, and transport (21). *APX6* protects *Arabidopsis* seeds to properly execute the germination program through the modulation of ROS crosstalk with hormonal signals (30). In our *GhAPXs* suppression lines, auxin homeostasis was altered during S.E. compared with wild type (Fig. 7), suggesting that *GhAPX-*mediated ROS signaling interacts with auxin signaling in this developmental process.

Reduced catalase activity results in the accumulation of H_2_O_2_ and attenuation of auxin levels at high light intensities (70). Mutation of the mitochondrial *FtSH4* gene significantly elevated H_2_O_2_ content and decreased the concentration of IAA and expression of auxin-related genes (71). These examples are consistent with attenuated levels of free IAA and IAA metabolites observed after H_2_O_2_ treatment (Fig. 7*A*). Localized auxin accumulation could cause ROS generation and increase oxIAA formation by IAA oxidase to attenuate auxin signaling (72). However, the content of oxIAA was dynamically altered during the dedifferentiation process by H_2_O_2_ treatment (Fig. 7*A*). It might be a complicated feedback regulation of ROS and auxin during the S.E. process.

The expression of some auxin-related genes, including those involved in auxin metabolism, auxin signaling and auxin transport, was elevated after H_2_O_2_ treatment (Fig. 7*C*), a result that is not consistent with the negative effects of ROS on the expression of at least some auxin-related genes (71). A detailed analysis of the transcriptional regulation of the auxin signaling pathway through apoplastic ROS revealed that ROS transiently decreases the expression of auxin-related genes (31). The transcript levels of *GH3* genes, including some *AUX/IAA* and *ARF* genes, was both up- and down-regulated through ROS, with no consistent trend, and the expression of the auxin-responsive marker gene *HAT2* was differentially altered during O_3_ treatment (31). Furthermore, the expression of several *AUX/IAA* and *SAUR* genes and the auxin efflux carrier *PIN3* was regulated through ROS or auxin in an inverse pattern (31), indicating the dynamic and intricate regulation mechanisms of ROS on auxin-related genes.

In agreement with this, the expression of several auxin-related genes was differentially altered in *GhAPXs* suppression lines (Fig. 7*E*). Expression levels of auxin homeostasis-related genes in *GhAPXs* suppression lines were different from those observed with H_2_O_2_ treatment, likely reflecting the different roles of specific *GhAPXs*. It has been reported that the auxin-regulated counterbalance of APX1 with S-nitrosylation/denitrosylation to regulate APX1 activity also regulates root development in *Arabidopsis* (28). The level of auxin homeostasis has been found to increase in *APX6*-deficient *Arabidopsis* seeds, further suggesting ROS signal crosstalk with hormone signaling (30).

We found that auxin homeostasis was also perturbed during S.E. after DPI treatment (Fig. 7*A*). AtrbohD and AtrbohF have previously been shown to negatively regulate the auxin response in the root tip in the presence of ABA (32). Knocking down the expression of *rbohB* has shown to decrease ROS production and elevated the expression of some auxin signaling-related genes, suggesting that the generation of ROS through *rboh* genes impacts root development via auxin signaling (73). Consistent with this, the expression of auxin response genes, such as *GhAUX/IAA* and *GhSAUR*, and the auxin polar transport genes *GhABCB19*, *GhLAX1*, *GhPILS2*, *GhAUX1,* and *GhPIN1*, was increased after DPI treatment compared with normal control tissues after culture for 15 d (Fig. 7*C*).

**Fig. 8.**
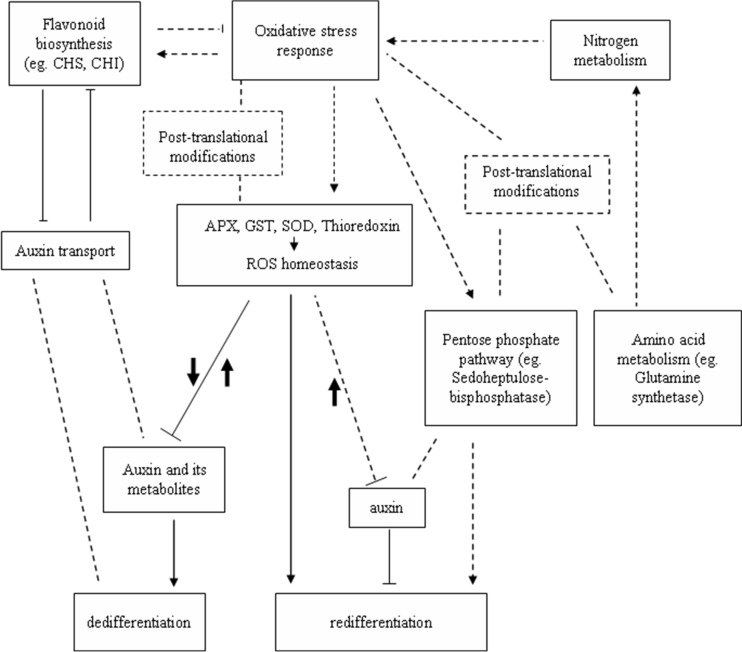
**A general scheme showing proposed regulation mechanisms in cotton S.E**. Bold black arrows up or down represent the contents of ROS were elevated or declined, respectively. Arrows indicate positive regulation. T-bars indicate negative regulation. Dotted lines without arrow indicate there were connections between the two aspects. Dotted lines with arrows indicate hypothetical regulation.

Interestingly, the application of auxin partially reversed the inhibitory effects of DPI and H_2_O_2_ (Figs. 7*E* and 7*F*), and the perturbation of dedifferentiation through 2,3,5-triiodobenzoic acid was partially recovered by application of moderate H_2_O_2_ concentrations during late-stage dedifferentiation (Fig. S7). These results further indicate an interaction between ROS and auxin in cotton S.E. In general, for the SE process correlated with oxidative stress, moderate ROS was necessary for dedifferentiation, while excess ROS inhibited the dedifferentiation process, indicating ROS homeostasis was important for dedifferentiation, which was maintained by the activity of ROS-related proteins, such as APX, GST, SOD, and thioredoxin. ROS generation inhibited or enhanced both attenuated free IAA content and altered auxin metabolism, which then perturbed the dedifferentiation process. ROS homeostasis was dynamically integrated with auxin homeostasis to regulate dedifferentiation process, while enhanced ROS production promoted redifferentiation, which might be due to the attenuated free IAA (Fig. 8). On the other hand, flavonoid biosynthesis might be coupled with oxidative stress to modulate ROS homeostasis and function in auxin transport inhibition to regulate dedifferentiation. Oxidative stress might also activate the pentose phosphate pathway, which then stimulates redifferentiation. Amino acid metabolism might be involved in the SE process through modulating nitrogen metabolism, which connects with oxidative stress response, to regulate the SE process. Several enzymes involved in the pentose phosphate pathway and amino acid metabolism as well as stress-related proteins are targets for posttranslational modifications; therefore, the posttranslational modification mechanism might be involved in metabolic control of S.E. process (Fig. 8). Further and deeper studies are needed to confirm whether the complex interaction between ROS and auxin is integrated with other signal networks to regulate the initiation and development of cotton S.E.

## Footnotes

^1^ The abbreviations used are:

ASA: ascorbic acid
2-DE: two-dimensional gel electrophoresis
BSO: buthionine sulfoximine
CEs: cotyledon embryos
CHAPS: 3-[(3-cholamidopropyl) dimethylammonio]-1-propanesulfonate
DEPs: differentially expressed proteins
DMSO: dimethyl sulfoxide
DPI: diphenyleneiodium
DTT: dithiothreitol
EC: embryogenic callus
GEs: globular embryos
Gh: *Gossypium hirsutum*
GSH: reduced glutathione
NEC: nonembryogenic callus
oxIAA: 2-oxoindole-3-acetic acid
PMSF: phenylmethanesulfonyl fluoride
qRT-PCR: quantitative real time PCR
ROS: reactive oxygen species
SDS: sodium dodecyl sulfate
SE: somatic embryogenesis
SSP: sample spot protein
TCA: trichloroacetic acid
TEs: torpedo embryos
Della: DELLA proteins are a class of transcription factor which contain a conserved DELLA domain, and act as key repressors of gibberellin (GA) signaling.
